# Relationship between Reproductive Allocation and Relative Abundance among 32 Species of a Tibetan Alpine Meadow: Effects of Fertilization and Grazing

**DOI:** 10.1371/journal.pone.0035448

**Published:** 2012-04-19

**Authors:** Kechang Niu, Bernhard Schmid, Philippe Choler, Guozhen Du

**Affiliations:** 1 Department of Biology, Nanjing University, Nanjing, China; 2 MOE Key Laboratory of Arid and Grassland Ecology, Lanzhou University, Lanzhou, China; 3 Institute of Evolutionary Biology and Environmental Studies, Zurich University, Zurich, Switzerland; 4 Laboratoire d'Ecologie Alpine, UMR-CNRS 5553 and Station Alpine J. Fourier, CNRS -UMS 3370, Université de Grenoble, Grenoble, France; University of Utah, United States of America

## Abstract

**Background:**

Understanding the relationship between species traits and species abundance is an important goal in ecology and biodiversity science. Although theoretical studies predict that traits related to performance (e.g. reproductive allocation) are most directly linked to species abundance within a community, empirical investigations have rarely been done. It also remains unclear how environmental factors such as grazing or fertilizer application affect the predicted relationship.

**Methodology:**

We conducted a 3-year field experiment in a Tibetan alpine meadow to assess the relationship between plant reproductive allocation (RA) and species relative abundance (SRA) on control, grazed and fertilized plots. Overall, the studied plant community contained 32 common species.

**Principal Findings:**

At the treatment level, (i) RA was negatively correlated with SRA on control plots and during the first year on fertilized plots. (ii) No negative RA–SRA correlations were observed on grazed plots and during the second and third year on fertilized plots. (iii) Seed size was positively correlated with SRA on control plots. At the plot level, the correlation between SRA and RA were not affected by treatment, year or species composition.

**Conclusions/Significance:**

Our study shows that the performance-related trait RA can negatively affect SRA within communities, which is possibly due to the tradeoffs between clonal growth (for space occupancy) and sexual reproduction. We propose that if different species occupy different positions along these tradeoffs it will contribute to biodiversity maintenance in local communities or even at lager scale.

## Introduction

Understanding why some species are common and others are rare at a particular site is one of the most difficult challenges in biology [1]. Mechanisms potentially explaining species relative abundance (SRA) distributions in communities include niche-based deterministic and neutral stochastic ones. From a classical niche perspective, SRA distributions within communities are driven by tradeoffs among performance-related traits of co-occurring species [2], [3] such as traits related to competitive vs. colonization ability [4]–[6]. However, ecologists have often failed to find strong correlations between species traits and SRA [7], [8]. This supported a new neutral perspective asserting that community assembly may largely be driven by random drift of dispersal-limited species in and out of the community, regardless of their traits and ecological differences among them [9]–[11]. The neutral perspective offered a convenient null model against which other perspectives could be compared and thus triggered renewed interest into the potential role of traits and tradeoffs in community assembly [12]–[15].

Previous studies on trait–abundance relationship mainly focused on the link between plant functional traits and species presence/absence [7], [16]–[18]. The complexity of trait interactions (e.g. tradeoffs), trait syndromes and the environmental context make it difficult to find functional traits with consistently strong relations to performance. This may be one of the reasons why a linkage between plant functional traits and species abundance often could not be demonstrated in field experiments [7], [8], [19]. However, the link should be particularly strong if plant traits are closely related to plant performance also called performance traits [13]. Such traits not only should explain presence vs. absence [20], but also the level of abundance of particular species (SRA) in a local community [12], [21]. Alternatively, if community structuring is driven by random drift of ecologically equivalent species, significant trait–abundance relationships would not be expected [10], [22].

A trait with a particularly high potential to affect plant performance is biomass allocation (e.g. allocation to vegetative growth or to reproduction). Allocation to vegetative growth should increase the competitive ability and the potential of a species to become more abundant in an occupied site, whereas reproductive allocation (RA) should increase a species' potential to colonize new sites. Assuming a tradeoff between allocation to growth and reproduction [23]–[25], it is therefore expected that, locally and over short time scale, RA should be negatively correlated with SRA [23], [26]–[28] whereas at a larger spatial scales and over longer time scales RA should be positively correlated with SRA [13], [29]. Similarly, the tradeoff between asexual ( = clonal growth) and sexual reproduction is also related to species performance and hence to SRA. High allocation to sexual reproduction should enhance species dispersal and colonization ability, while a high allocation to vegetative production should increase species abundance in local communities. Thus, negative RA-SRA relationships are expected if dominant species are able to reproduce both sexually and vegetatively. Moreover, variation in species performance and SRA can result from phylogenetic effects [30]–[32] or related Janzen–Connell effects [33]–[35], which in turn can be associated with related to reproductive strategy [36], [37].

The relationship between RA and SRA at the local scale may further depend on the availability of light and soil resources. If resources are scarce, preferential allocation of biomass to vegetative growth (shoots and roots) may be particularly important to maintain site occupancy and therefore reduced RA. In that case, RA and SRA should be negatively correlated. If light availability increases because of grazing or increased soil nutrient availability following fertilizer application, then a higher RA should be possible without negative effects on SRA. Furthermore, grazing may also increase the possibility for colonizing new microsites by dispersing seeds, thus providing an advantage to species with high RA [38]–[40]. Therefore, in the present study we assessed the relationship between RA and SRA at the local, i.e. plant community scale in alpine meadows of the Tibetan Plateau. These meadows exhibit a high diversity with about 30 to 50 plant species per 0.5×0.5 m quadrat. Previous studies have shown the strong sensitivity of these ecosystems to changes in soil fertility and grazing pressure [41]–[43]. A 3-year long experiment has been conducted in plots where we controlled for grazing activity and fertilizer application to test the above predictions. We asked the following questions: i) is RA negatively correlated with SRA in control plots and ii) is this negative correlation not observed in grazed and fertilized plots?

## Methods

### Ethics Statement

No permits were required to carry out this study.

### Study site

The experiment was conducted in the MaQu branch of the Research Station of Alpine Meadow and Wetland Ecosystems of Lanzhou University (N33°59′, E102°00′, altitude 3500 m a.s.l). The site is located in the MaQu County which belongs to the eastern part of the Tibetan Plateau, Gansu province, China. The mean annual temperature is 1.2°C, ranging from −10°C in January to 11.7°C in July. The mean annual precipitation for the period 1975–2010 was 620 mm, occurring mainly during the short, cool summer. The annual duration of cloud-free solar radiation is about 2580 h. For further details about the field site see [19], [41].

### Experimental design

A 13 ha flat, alpine grassland was enclosed within 58 ha of fenced grassland in October 1999. Grazing was allowed within the enclosure only during the non-productive winter months. Outside of the enclosure (45 ha), vegetation was moderately grazed by 110 yaks and 2,200 sheep during all months except for 40 days between July and mid-August when the animals were moved to high-altitude pastures [19].

In late May 2004, thirty 5×8 m plots, separated by 2 m, were established within the fenced site. We randomly allocated control, low and high fertilizer-addition treatments (30 and 60 g fertilizer, respectively, per square meter) to plots and replicated each of these treatments ten times. A slow-release, pelletized fertilizer (30 g/m^2^ of (NH_4_)_2_HPO_4_, 18% N and 46% P) was hand-broadcast once annually at the end of May during drizzly days to avoid the need for watering [41]–[43]. Outside the enclosure, at a distance of 300 m from the fertilized plots, ten 5×8 m plots, separated by 2 to16 m, were randomly established for the grazing treatments.

Each plot was divided into two parts: a 5×5 m subplot for measurements of plant traits and a 5×3 m subplot for community monitoring. Aboveground biomass production of the total plant community and the availability of light and soil resources for the 40 plots have been reported previously [41].

### Species abundance measurements

In the middle of September 2004, 2005 and 2006, a 0.5×0.5 m quadrat was harvested from the 5×3 m subplot of each plot. Harvested quadrats were located at different places each year. The number of individuals was counted for each species before clipping. For clonal plants, the term individual refers to ramets [42]–[44]. These are equivalent to tillers in grasses and rosettes or rooting branches in forbs. Aboveground green parts (stem and leaves) were sorted by species and brought to the laboratory.

### Biomass allocation measurements

Based on previous studies, we chose 32 common species (Figure S1) for measuring biomass allocation as well as collecting seeds. These species accounted for 85–95% of the aboveground biomass and 80–90% of the vegetation cover of the total plant community. The species were split into two functional groups: forbs (including legumes) and graminoids. Individuals were sampled in September 2004, 2005 and 2006. The harvesting schedule took account of the different phenologies of the species, i.e., species were sampled at their fruiting time. Only aboveground parts were collected because the sampling of individual root systems was deemed impossible in this dense meadow. We randomly sampled 2–3 adult individuals of each species in the 5×5 m subplot of each plot, so as to obtain nearly 30 individuals for each treatment. In grazed plots, we selected individuals that were not injured by grazing. In the laboratory, individuals were split into stems, leaves and reproductive parts (flowers and fruits). Samples were dried at 80°C and weighed to the nearest 10^−4^ g. The individuals sampled in 2004 from grazed plots were discarded because too many of them were damaged.

### Measurements of seed size

We also collected approximately 500 mature seeds from 20–30 individuals of each of the 32 species on the fenced control plots over the three years. We deposited the seeds in envelopes and spread them on tables in the laboratory (approximately 15°C) until they were dry. Three replicates of 100 dried seeds were weighed for each species to measure seed mass per 100 seeds.

### Data analysis

Species relative abundance (SRA) was defined as the number of individuals of a given species divided by the total number of individuals in each 0.5×0.5 m quadrat. There were thus up to 10 SRA values per species per treatment. We calculated the individual reproductive allocation (RA = biomass of reproductive parts/aboveground biomass (IB)), stem allocation (SA = biomass of stems/IB) and leaf allocation (LA = biomass of leaves/IB). Species biomass allocation was calculated as the mean of the 25–30 individual biomass allocation values per species and treatment [42].

Firstly, we examined the relationship between RA (or IB) and log-transformed SRA by calculating Spearman rank correlations at the treatment level, i.e. correlating species RA with SRA (averaged over the 10 replicate quadrats within each treatment) for each treatment and year separately. For the control treatment, we also examined the relationship between seed size and SRA. Secondly, to examine whether RA correlated with SRA on plot level, we used a linear mixed-effects model with log-transformed SRA as dependent variable and RA, year, treatment and plant functional group as fixed explanatory terms and plot and species as random explanatory terms. Interactions between fixed terms were not significant and we thus excluded them in our final analysis. Finally, to test the effects of the number of replicates per species and of phylogenic relationships between species on the correlations, we used bootstrapping analyses with 10,000 simulations and phylogenetically independent contrasts (PICs) [45], [46]. For the mixed models, we used the nlme [47] and lme4 functions of lme4 package [48] developed for the statistical software R [49]. Based on the published phylogenetic supertree of angiosperm families and APG III [50], [51], we built a phylogenetic tree of present species in this study with Phylomatic [52] and Phylocom [53], and tested phylogenetically independent contrasts (PICs) with the ape and ade4 R packages [54], [55].

## Results

**Figure 1:**
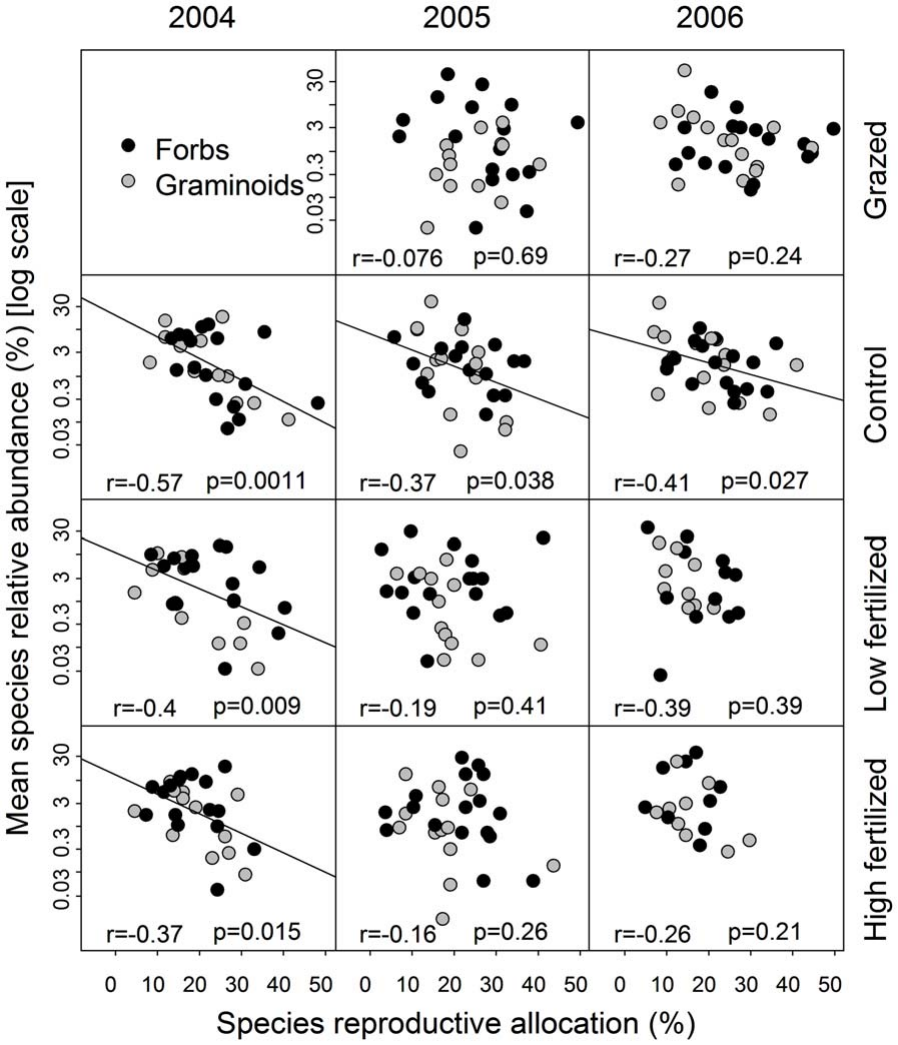
The relationships between species relative abundance (SRA) and reproductive allocation (RA) in control, fertilized and grazed plots. The dots indicate means of 25–30 individual RA for each species and its mean SRA over 10 quadrats. r and p values were estimated from Spearman rank correlations.

**Figure 2:**
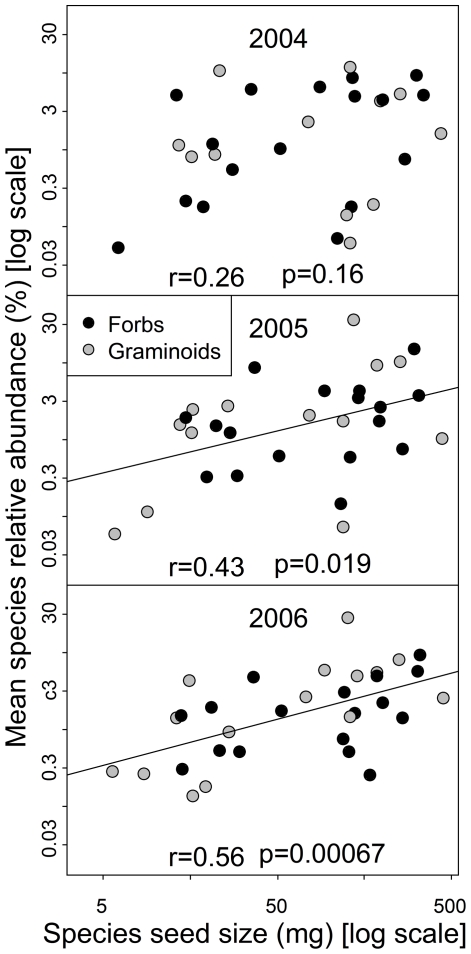
The relationships between species relative abundance (SRA) and seed size in control plots. The dots indicate seed mass per 100 seeds (3 replicates) for each species and its mean SRA over 10 quadrats. r and p values were estimated from Spearman rank correlations.

At the treatment level, we found that the mean RA of species was significantly negatively correlated with mean SRA in all three years in control plots. In fertilized plots, the correlation was still significantly negative during the first year but had disappeared in the second and third year. There was no significant relationship in grazed plots (Figure 1). The negative correlations strengthened when we used bootstrapping simulation and PICs (not shown). Mean SRA was positively correlated with the mean seed size of species in control plots for two years (Figure 2).

On plot level, results of the mixed-model analyses showed that SRA was negatively affected by RA (F = 7.48, p<0.01) and that the effect varied significantly between years (F = 5.16, p<0.01). Treatments did not significantly differ in the mixed-model analyses (F = 0.71, p>0.5) but graminoids had larger SRA than forbs (F = 5.08, p<0.05). The variance component for species was large (0.314±0.085), indicating strong differences in SRA between species within functional groups.

There were no significant correlations between mean SRA and mean species aboveground biomass in all plots (Figure S2). Mean SRA significantly positively correlated with mean species leaf allocation in control and grazed plots (Figure S3). Finally, mean SRA tended to be negatively correlated with mean species stem allocation, but this was only significant in 2006 (Figure S4).

## Discussion

### Reproductive allocation and the balance between competition and colonization ability

A potential key driver maintaining biodiversity within and between communities are tradeoffs arising from the need of organisms to balance their allocation of limited energy (biomass) among growth, reproduction and defense [56]–[58]. The tradeoff in biomass allocation results from physical and chemical constraints during the life history of organisms [57], [59]. Typically, plants allocate more biomass to roots, leaves and stems than to reproduction when competition for water, nutrient and light is strong [23], [26]. In contrast, the possibility of colonizing new microsites increases with allocation to sexual reproduction [60]. Furthermore, plants have to balance their production of seeds along a tradeoff between many small vs. few large seeds [58]; and typically plant species or genotypes with high RA produce many small seeds to increase their colonization ability [58], [61], [62].

In the Tibetan alpine meadows studied here, we previously observed that plant species allocated more biomass to leaves at the expense of RA under increased light competition in fertilized plots [42], [63], whereas, they often increased RA at the expense of stem or even leaf growth in grazed plots [64]. Correspondingly, biomass allocation to clonal growth increased under fertilization whereas it decreased under grazing [65]. Based on these findings, species with smaller RA were expected to have higher competitive ability due to either larger root, stem, and leaf allocation or increased clonal growth, whereas species with higher RA were expected to have lower competitive ability; additionally they were expected to produce more (smaller) seeds to increase their colonization ability. However, we could not directly test the colonization ability in the present study.

### Reproductive allocation and species relative abundance

Consistent with these predictions, we found that increased RA had a negative influence on the individual abundance of given species, suggesting that species which invested more into sexual reproduction and colonizing ability had lower competition ability and thus could not maintain high abundance within a local site and community. In contrast to RA, functional traits such as specific leaf area or mature height of species were not correlated with SRA (data not shown). This may be because performance-related traits can be related to several functional traits in different ways such that no single one of them can predict performance in a particular environment. It has been suggested that, compared to functional traits, performance-related traits should be more tightly linked to species abundance [13], [29]. As our results suggest, biomass allocation as a key performance-related trait and specifically RA as indicator of an among-species tradeoff between growth and sexual reproduction could determine the pattern of SRA within local sites, and influence community structuring in response to environmental factors such as fertilization and grazing. The negative correlation between RA and SRA and the positive correlation between leaf allocation and SRA are consistent with the hypothesis of a tradeoff between growth and sexual reproduction.

The positive correlation of leaf allocation with SRA in our study suggests that light competition was an important driver for community structuring [66]–[71]. In contrast, stem allocation in the studied species was presumably less important for light competition, because most species only carried flowers and fruits on their stems but not leaves (tillers and rosettes as typical growth forms) [42]. This could explain the observed weak negative correlation between stem allocation and SRA. Furthermore, we should mention that differences in SRA were not simply due to different overall plant sizes between species, because SRA was not correlated with mean species aboveground biomass (Figure S2). Finally, the positive relationship between SRA and mean seed size of species supports the idea of a tradeoff between competition and colonizing ability that caused species with high RA to be locally less abundant than species with low RA and consequently high leaf allocation. Such a relationship between seed size and the competition–colonization tradeoff has often been documented [72]. Theoretically, root allocation should be positively correlated with SRA under competition for limiting soil resources, but it was not possible to assess this relationship in the dense meadows of our study site because roots of different species intermingle too much.

In addition, as discussed above, the tradeoff between growth and sexual reproduction can also result in a negative RA–SRA pattern when dominant (and abundant) species reproduce primarily by clonal growth and rare species recruit from seeds. In our site, the dominant species (*Kobresia capillifolia*) reproduces mainly by clonal growth, and many other abundant grass species recruit by both sexual reproduction and clonal growth, of which seed production often dominates. Then, if the tradeoff between clonal growth and sexual reproduction would determine the negative RA–SRA relationship, the latter should disappear when sexual reproduction in these species becomes rare. However, the correlation was still significant when we removed some species with clonal growth or even all of graminoids. Moreover, the PIC analysis showed that when we deducted the phylogenetic effect the negative RA–SRA relationship became even stronger. This suggested that the negative RA–SRA correlation was not due to the fact that the observed species occupied different positions along the phylogenetic tree. In short, these inferences suggest that the negative RA–SRA relationship may not result from a simple tradeoff between clonal growth and sexual reproduction or phylogenetic effects. But we still need more comprehensive research to distinguish the role of these processes in determining patterns of RA–SRA relationships.

### Influence of grazing and fertilization on the SRA–RA relationship

We suggested in the Introduction that strong competition for light and soil resources could be responsible for negative correlations between SRA and RA, because under these circumstances species should invest more into growth then into reproduction to keep a site occupied at high abundance. Indeed, we found the strongest evidence for a negative relationship between SRA and RA in fenced, unfertilized plots. In grazed or fertilized plots the correlation between SRA and RA was, however, weak and often non-significant.

In grazed plots both RA and SRA of most species increased (points moved toward the top right corner of graphs in Figure 1; see also [64]). In fertilized plots, both RA and SRA of many species deceased [42], [63]. These results suggest that if tradeoffs in (clonal) growth and reproduction drive SRA, the negative SRA–RA correlations may weaken after grazing (reduced light competition). In contrast, fertilization might have made competition for light so strong that species with large RA (generally forbs which had overall lower SRA in the analysis on plot level) were lost due to competitive exclusion, thus shortening the range of species RAs that could be compared along the x-axis of the relationship with SRA [42], [43].

In conclusion, our results support the hypothesis that patterns of SRA in Tibetan alpine meadows are not the result of neutral processes but rather due to differences in species' positions along tradeoffs between (clonal) growth and sexual reproduction. The balance between competition and colonizing ability may structure these plant communities and explain the large biodiversity within and among local communities.

## Supporting Information

Figure S1 **Phylogenetic tree of the 32 investigated species.**
(DOC)

Figure S2 **Correlations between species relative abundance (SRA) and individual above-ground biomass in control, grazed and fertilized plots.** The dots indicate means of 25–30 individual above-ground biomass for each species and its mean SRA over 10 quadrats. r and p values were estimated from Spearman rank correlations.
(DOC)

Figure S3 **Correlations between species relative abundance (SRA) and leaf allocation (LA) in control, grazed and fertilized plots.** The dots indicate means of 25–30 individual LA for each species and its mean SRA over 10 quadrats. r and p values were estimated from Spearman rank correlations.
(DOC)

Figure S4 **Correlations between species relative abundance (SRA) and stem allocation (SA) in control, grazed and fertilized plots.** The dots indicate means of 25–30 individual SA for each species and its mean SRA over 10 quadrats. r and p values were estimated from Spearman rank correlations.
(DOC)
