# Supplementary material for: Relationship between Reproductive Allocation and Relative Abundance among 32 Species of a Tibetan Alpine Meadow: Effects of Fertilization and Grazing
Source: PLoS One. 2012 Apr 19;7(4):e35448. doi: 10.1371/journal.pone.0035448 (PMC3334899; doi:10.1371/journal.pone.0035448)
Supplement: Figure S1 — Phylogenetic tree of the 32 investigated species. (DOC) [file pone.0035448.s001.doc]

**Figure S1** Phylogenetic tree of the 32 investigated species.

*Allium sikkimense*

*Agrostis hugoniana*

*Agrostis micrantha*

*Elymus nutans*

*Festuca sinensis*

*Koeleria litvinowii*

*Poa chalarantha*

*Poa pratensis*

*Roegneria nutans*

*Stipa aliena*

*Kobresia capillifolia*

*Anemone obtusiloba*

*Anemone rivularis*

*Delphinium kamaonense*

*Ranunculus tanguticus*

*Ranunculus tanguticus* var. *nematolobus*

*Ajania tenuifolia*

*Ligularia virgaurea*

*Saussurea hieracioides*

*Saussurea nigrescens*

*Taraxacum maurocarpum*

*Bupleurum smithii*

*Carum carvi*

G*entianopsis paludosa*

*Halenia elliptica*

*Swertia bimaculata*

*Veronica eriogyn*

*Euphorbia micractina*

*Tibetia himalaica*

*Medicago ruthenica*

*Potentilla saundersiana*

*Geranium pylzowianum*
