# Supplementary material for: Relationship between Reproductive Allocation and Relative Abundance among 32 Species of a Tibetan Alpine Meadow: Effects of Fertilization and Grazing
Source: PLoS One. 2012 Apr 19;7(4):e35448. doi: 10.1371/journal.pone.0035448 (PMC3334899; doi:10.1371/journal.pone.0035448)
Supplement: Figure S2 — Correlations between species relative abundance (SRA) and individual above-ground biomass in control, grazed and fertilized plots. The dots indicate means of 25–30 individual above-ground biomass for each species and its mean SRA over 10 quadrats. r and p values were estimated from Spearman rank correlations. (DOC) [file pone.0035448.s002.doc]

**Figure S2** Correlations between species relative abundance (SRA) and individual above-ground biomass in control, grazed and fertilized plots. The dots indicate means of 25–30 individual above-ground biomass for each species and its mean SRA over 10 quadrats. r and p values were estimated from Spearman rank correlations.
