# Supplementary material for: Relationship between Reproductive Allocation and Relative Abundance among 32 Species of a Tibetan Alpine Meadow: Effects of Fertilization and Grazing
Source: PLoS One. 2012 Apr 19;7(4):e35448. doi: 10.1371/journal.pone.0035448 (PMC3334899; doi:10.1371/journal.pone.0035448)
Supplement: Figure S3 — Correlations between species relative abundance (SRA) and leaf allocation (LA) in control, grazed and fertilized plots. The dots indicate means of 25–30 individual LA for each species and its mean SRA over 10 quadrats. r and p values were estimated from Spearman rank correlations. (DOC) [file pone.0035448.s003.doc]

**Figure S3** Correlations between species relative abundance (SRA) and leaf allocation (LA) in control, grazed and fertilized plots. The dots indicate means of 25–30 individual LA for each species and its mean SRA over 10 quadrats. r and p values were estimated from Spearman rank correlations.
